# Facilitating the transfer of care from secondary to primary care: a scoping review to understand the role of pharmacists in general practice

**DOI:** 10.1007/s11096-023-01547-3

**Published:** 2023-03-15

**Authors:** Faiza Yahya, Hamde Nazar, Clair Huckerby, Muhammad Abdul Hadi

**Affiliations:** 1 Our Health Partnership, First Floor, 1856 Pershore Road, Cotteridge, Birmingham, B30 3AS UK; 2 School of Pharmacy, Newcastle University, King George VI Building, Newcastle upon Tyne, NE1 7RU UK; 3 College of Pharmacy, QU Health, Qatar University, 2713 Doha, Qatar

**Keywords:** General practice, Medication review, Pharmacists, Primary care, Secondary care, Transfer of care

## Abstract

**Background:**

Limited published research exists on the role of pharmacists in general practice (primary care pharmacists) in facilitating post-hospital discharge care of patients.

**Aim:**

To summarise and map the nature and extent of current evidence on the role of primary care pharmacists in facilitating patient discharge from secondary to primary care and to inform future practice and research.

**Method:**

Six electronic databases were systematically searched from inception to March 2022 for studies published in the English language that described and/or evaluated primary care pharmacist-led interventions for patients following hospital discharge. Grey literature and reference lists of included studies were also searched. Two authors independently screened articles for selection. A structured, pilot-tested form was used for data extraction.

**Results:**

Twenty articles were included. The majority of studies (n = 17; 85%) were conducted in the USA. The most frequently reported intervention made by primary care pharmacists were medication reviews (n = 18) and medication reconciliation (n = 16). Studies have demonstrated the emerging roles of pharmacists involving collaboration with other healthcare professionals, review of laboratory monitoring, referrals, and follow-up. A wide range of outcomes such as the impact on hospital readmission rates, healthcare utilisation and reduction of potential adverse drug events were reported.

**Conclusion:**

Pharmacists in general practice can offer a range of interventions in facilitating transfer of care of patients from secondary to primary care with positive patient and healthcare utilisation outcomes. However, more rigorous research evidence is required to establish the effectiveness, generalisability, acceptability, and sustainability of these services.

**Supplementary Information:**

The online version contains supplementary material available at 10.1007/s11096-023-01547-3.

## Impact statements

- Pharmacists working in general practice can offer a range of interventions to facilitate transitions of care.
- Although the evidence suggests that pharmacists can have a positive impact on facilitating transitions of care, limited research evidence is available from outside the USA.

## Introduction

Research suggests that patients will have, on average, 4.4 drug changes upon hospital discharge [1] and 50% of adult patients experience medication errors or unintentional medication discrepancies during this transition [2]. This is a well-known risk factor for medication-related harm, a significant burden on healthcare systems globally and identified as a national and global priority area for quality improvement [3, 4]. It is estimated that 74% of potentially avoidable costs are attributed to medication prescribed at hospital discharge [5, 6] and medication being one of the three main causes of potentially preventable 30-day hospital re-admissions [7]. Hospital discharge is often a very disruptive time for patients with many factors contributing to poor understanding and adherence of medications [8, 9], increasing the risk to patient safety.

Much consideration in terms of research, policy and practice has been invested to achieve safer transition of care such as implementing digital transfer of information [3, 10, 11], and recognising the need for timely intervention pathways post-discharge [12]. Nonetheless, medication management during transitions remains a significant problem, especially for the most vulnerable patients [13, 14].

Pharmacists in collaboration with patients, other healthcare professionals and/or carers [15], can play an important role to support safe and effective medicines-related continuity of care [15, 16]. Many studies have evaluated the impact of pharmacist interventions such as medicines reconciliation [16–18], and medication reviews [19], in hospital and community pharmacy settings. Many systematic reviews and meta-analyses to date have reported limitations with poor and inconsistent descriptions of pharmacist interventions in such studies [17–21]. Some systematic reviews on medicines reconciliation in the primary care sector do not differentiate between the role of the primary care pharmacist (PCP) in a general practice setting or in a community pharmacy setting [17]. This nuance of where particular packages of care are delivered and by whom they are delivered are important pieces of contextual information about intervention implementation and delivery and crucial to better understanding of how, and why an intervention works or not [22].

Clinical pharmacy practice is evolving at pace around the world [23, 24]. Primary care is experiencing unprecedented pressures in patient health-seeking behaviours but also in managing a backlog following the COVID-19 pandemic [25, 26]. There is an accelerated training and mobilisation of pharmacists into general practice to increase capacity in this sector in the UK [27–29]. Healthcare systems continue to identify roles and responsibilities for these pharmacists and their involvement in hospital discharge care is highly likely [28, 29].

This scoping review sought to explore the role of PCPs in the transfer of care of patients from hospital back home to ensure ongoing policy, practice and research is informed by current evidence. PCPs for the purpose of this review are defined as pharmacists working in a general practice setting, not in community pharmacy or hospital settings.

## Aim

To map the nature and extent of current evidence on the role of PCPs in facilitating patient discharge from secondary to primary care.

### Objectives

- To identify literature that investigates the role of primary care pharmacists in patients’ post-hospital discharge care.
- To identify what study designs and types of interventions carried out by PCPs and what outcomes have been researched in post-discharge care.
- To synthesise research evidence and identify gaps in literature to inform future practice and research.

## Method

A scoping review was identified as the most appropriate methodology to map the extent and nature of research undertaken on the role of PCPs post-hospital discharge and identify knowledge gaps to inform future research [30, 31].

This scoping review was conducted in accordance with the Joanna Briggs Institute (JBI) methodology for scoping reviews [32] and a pre-defined published protocol [33].

### Eligibility criteria

This scoping review aimed to include all published primary studies using both observational (e.g., case control, cohort) and experimental (e.g., randomised controlled clinical trials, quasi-experimental) methodologies. Articles were excluded if they primarily involved interventions for hospital in-patients, outpatient clinics, or home medication reviews. Studies involving paediatric and oncology patients were also excluded. Conference abstracts, protocols and case reports were excluded as these were deemed to provide limited evaluative benefit. The eligibility of studies was guided by the Population, Concept and Context (PCC) mnemonic as recommended by the JBI guidelines for scoping reviews [32].

#### Participants

Adult patients (aged 18 or above) recently discharged from hospital and had an intervention by a PCP, regardless of the outcome assessed, the profile of patients included or the clinical diagnosis on admission.

#### Concept

Articles must report interventions led by PCP for patients recently discharged from hospital.

#### Context

This review aimed to summarise PCP interventions carried out in a primary care/ general practice setting.

### Search strategy

A comprehensive literature search was undertaken (Supplementary File 1) with the support of a medical research librarian and followed a three-step search strategy as per JBI guidelines [34]. Firstly, an initial limited search on the topic was undertaken in MEDLINE, Cochrane Library and Cumulated Index to Nursing and Allied Health Literature (CINAHL Plus). The text words contained in the titles and abstracts and index terms of relevant articles were used to develop a full search strategy. Secondly, a full systemised search strategy was conducted using MEDLINE, EMBASE, PubMed, Cochrane central register of controlled trials (CENTRAL), Web of Science and National Institute for Health and Care Excellence (NICE) Evidence from their inception until March 2022. Several websites for relevant professional organisations were also searched for grey literature relevant to the topic. These included the Royal Pharmaceutical Society, General Pharmaceutical Council, Royal College of General Practitioners, Department of Health, the UK Faculty of Public Health, and the NICE websites. Finally, reference lists of included full texts were searched for relevant articles. No date limitations were set, however studies published only in the English Language were included. The search terms used have been detailed in supplementary file 2.

All identified articles were collated into an online research tool (Rayyan) [35] and duplicates were removed. Titles and abstracts were screened for eligibility by two independent reviewers (FY and MAH) before full-text screening was undertaken. Cases of disagreement were resolved via discussion or obtaining the full text. If it was still unclear if eligibility criteria were met, a third independent reviewer (HN) was contacted. For full texts that could not be retrieved, corresponding authors were emailed to request the full text of the article. If after this stage, the full text could not be retrieved then these articles were excluded. This was to ensure that only full papers were included for a comprehensive review. Reference lists of full texts included were then reviewed by one reviewer (FY) for further relevant articles.

### Data charting

Data were extracted and charted using standardised forms that were piloted on the initial 16 included articles and reviewed by the research team. The data extracted were mapped to answer the key objectives of this scoping review. During the data charting of relevant studies, an inductive content analysis approach was followed to collate the results [36]. Data extracted included: author, year, country, study design, population, concept, context, aims, methodology, outcomes and key findings, financial impacts reported, errors reported, collaborations reported, barriers/facilitators reported, and research gaps identified.

No quality or risk of bias assessment was performed as scoping reviews traditionally do not seek to assess the quality of evidence unlike systematic reviews but rather map what research has been undertaken [37].

### Data synthesis

A descriptive numerical and categorical analysis approach was undertaken to examine the extent, nature and distribution of papers included in the review. Key concepts relating to the review question(s) were collated in tabular format to identify themes and synthesise the findings. To classify and summarise the type of evidence available in this field and identify further research recommendations, the PAGER (Patterns, Advances, Gaps, Evidence for practice and Research recommendations) framework [38] was subsequently followed to enhance consistency and methodological rigour.

## Results

**Fig. 1:**
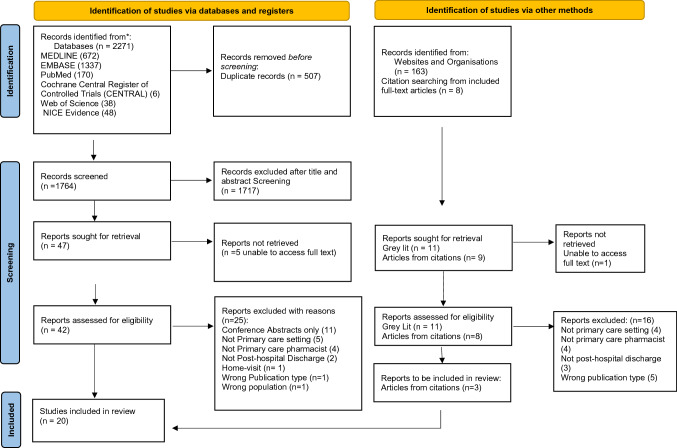
Preferred reporting items for systematic reviews and meta-analyses extension for scoping reviews (PRISMA-ScR) flow diagram

As shown in the PRISMA-ScR flow diagram (Fig. 1), the search retrieved 2271 publications. After removing duplicates (n = 915), titles and abstracts of 1764 articles were screened resulting in 42 full text articles being retrieved and reviewed for eligibility against inclusion and exclusion criteria. A grey literature search was undertaken as per the protocol and followed the same principles for screening. This yielded 11 articles for full-text screening and a citation-search of included full texts identified a further 8 studies, of which only 3 articles were suitable for inclusion. A total of 20 articles were subsequently included for the purpose of this review.

Although no date limitation was set, all 20 included studies were published within the last 10 years (between 2013 and 2021) with half of the studies (n = 10) [39–48] published in the last 2 years (after 2020). The majority of studies (n = 17) [39, 40, 42, 44, 46–58] were conducted in the United States of America (USA) and one each in the UK [45], Canada [41] and Australia [43].

Of the 20 included studies, half (n = 10, 50%) used observational methods [39–42, 44, 45, 48, 50, 52, 54], 40% (n = 8) were quasi-experimental studies [46, 47, 51, 53, 55–58] and only two were randomised controlled trials [43, 49]. Most studies (60%, n = 12) were retrospective in nature [40, 42, 44, 45, 47, 48, 50–54, 56]. Overall, the studies were mainly conducted at a single site (n = 12) [39, 40, 42, 44, 46–51, 56, 58].

Of the 20 included studies (see Table 1), 33.3% (n = 7) [42–44, 47, 50, 52, 53] specified a patient population discharged on five or more regular medicines and only 14.3% (n = 3) specified those on high-risk drugs [44, 50, 53]. Two studies used a Care Assessment Need (CAN) score to assess patients who were at high risk and targeted them for the intervention [40, 55]. Seven studies [40, 41, 47, 49, 56–58] included patients aged 60 years or older and 14.3% of studies (n = 3) [43, 50, 58] specified those cohorts who had a primary discharge diagnosis of congestive heart failure or exacerbation of the chronic obstructive pulmonary disease, as patients with these conditions have been identified as those who experience high readmission rates [43] (Table 2).

**Table 1:** Characteristics of included studies

Authors (First Author), Date	Country	Type of study design	Single /multi-centre	Population characteristics	Primary outcome(s)	Secondary outcomes(s)
Berquist et al. [39]	USA	Prospective Observational	Single	Adults aged 65 years and older who were discharged from the University of Colorado Hospital	Mean time per comprehensive medication review	Pharmacist interventions
Brauner et al. [40]	USA	Retrospective Observational	Single	Adults 18 years of age and over, with a Care Assessment Need (CAN) score of 90 or greater, and able to be scheduled in the pharmacist-run transitions of care clinic (face-to-face or telephone appointment) within 14 days of discharge	Frequency of 30-day, all-cause hospital readmission rates	Secondary outcomes were assessed for patients seen in the transitions of care clinic- medication-related recommendations
Cossette et al. [41]	Canada	Prospective Observational	Multi-site	Adults aged 65 years and older at risk of medication-related harm in three regions of Quebec, Canada,	Time to complete the interventions; the location of the interventions, whether the pharmacist met with the patient and/or caregiver; and follow-up	Number of Medication changes Hospitalisations and ED visits (exploratory and not subject to statistical testing)
Dellogono et al. [42]	USA	Retrospective Observational	Single	Adults aged 18 years or older with at least 5 medications	Medication Related Problems (MRP)- Type and Frequency	Pharmacist recommended interventions Potential and Adverse Drug Events
Fera et al. [50]	USA	Retrospective Observational	Single	Patients admitted to or discharged from the hospital with a diagnosis or history of COPD, heart failure, or polypharmacy Patients prescribed (on admission or at discharge) more than 9 medications, a high-risk medication, directly referred by primary care physician/care manager/via consultation, Patients with a readmission within 30 days of discharge	Acute care visits within 30 days	Hospital Consumer Assessment of Healthcare Providers and Systems (HCAHPS) scores and specific scores related to information provided about medications
Freeman et al. [43]	Australia	Prospective RCT	Multi-site	Adults (18 years or older) prescribed 5 or more long term regular medicines on discharge or had received a primary discharge diagnosis of CHF or COPD)	The rate of unplanned, all-cause, hospital re-admissions during the 12 months following discharge from the index admission	Re-admissions at 30 days, 3 months, and 6 months; ED presentations; Differences in costs between the intervention and normal care
Haag et al. [49]	USA	Prospective RCT	Single	Adults aged ≥ 60 years who were enrolled in the local Care transitions programme	To identify potentially inappropriate medications with the STOPP criteria and potential prescribing omissions using the START criteria	Assessment of medication utilization quality with the Medication Appropriateness Index (MAI) An assessment of healthcare resource utilization within 30 days of discharge,
Hawes et al. [51]	USA	Retrospective Quasi-experimental	Single	Adults aged 18 years or older, discharged from the academic institution’s FMIS, discharged to a community dwelling, had established primary care with an FMC provider, and attended a hospital follow-up visit within 30 days of discharge	Identifying Medication-Related Problems (using modified IMAP tool)	Healthcare Utilisation—hospital readmissions, as well as ED visits within 30 days and 60 days post hospital discharge, in those exposed to Pharmacist-enhanced care vs usual care
Herges et al. [53]	USA	Retrospective Quasi-experimental	Multi-site	Adults aged 18 years or older, had been dismissed from the hospital within 30 days and had at least 10 medications (including at least 1 high-risk medication) on their discharge summary list	Risk of readmission at 30 days	Risk of readmission at 60 days and 180 days after dismissal
Herges et al. [52]	USA	Retrospective Observational	Multi-site	Adults who were receiving at least one medication that put them at high risk for emergent hospital admission and at least 10 total medications	Readmission Rates (Hospital re-admissions at 30, 60, 180 days after the visit)	
Herges et al. [44]	USA	Retrospective Observational	Single	Adult patients recently discharged from a single institution, were 18 years of age or older, were on 10 or more medications at hospital discharge, including 1 or more “high readmission risk medications”	Cost Data; The total cost of care comprised costs associated with hospital readmissions, emergency department visits, and outpatient care utilization	
Kilcup et al. [54]	USA	Retrospective Observational	Multi-site	Adults discharged from any one of seven contracted hospitals and considered at higher risk for readmission and therefore needing transition care management	Rate of hospital readmission and financial savings	Financial savings The frequency of medication discrepancies for patients who received clinical pharmacist medication reconciliation
Ploenzke et al. [55]	USA	Prospective Quasi-experimental	Multi-site	Adults discharged from the institution's hospital or transitional care unit in the past 7 days, and classed as high risk (i.e. had a CAN score95th percentile)	Identification of Medication Related Problems and drug discrepancies	Pharmacist-mediated interventions; recommendations made to providers; time spent during visit; percentage of patients that participated in a visit; and number of care coordination referrals and/or consults for additional care placed
Shah C and Hough and Jani [45]	UK	Retrospective Observational	Multi-site	Patients (age not stipulated) discharged from hospital on medication between specified time period	Quality of discharge summaries—compliance with national minimum standards for medication-related information on dischargesummaries, (allergies, changes to medication regimen, minimum prescription standards and medicines reconciliation by the primary care team)	
Slazak et al. [46]	USA	Prospective Quasi-experimental	Single	Patients (age not stipulated) discharged from a hospital, skilled nursing facility, or rehabilitation facility to home	Pharmacist Transitions of care (TOC) Interventions and Acceptance Rates	Percentage of 30-Day Hospital Readmissions Odds Ratios of 30-day Hospital Readmissions between Intervention (Phase 1 & 2) and Usual Care Groups
Sorensen et al. [47]	USA	Retrospective Quasi-experimental	Single	Adults aged 65 and older, have two or more of the following risk factors, as identified using the UCLA Health electronic medical record (EMR): hospital readmission within the past 30 days and/or two or more admissions within the past 12 months; hospital length of stay greater than 10 days; 8 or more outpatient prescription medications; depression as a secondary diagnosis; mild cognitive impairment; two or more chronic conditions; and limited caregiver support, as determined by the referring source at the hospital, such as the care manager	The primary outcome measure was 30-day all-cause hospital readmissions	The secondary outcome measures were 60- and 90-day all-cause hospital readmissions, and 30-day ED visits
Stranges et al. [56]	USA	Retrospective Quasi-experimental	Single	Adults 60 years or older discharged from a large academic medical center	All-cause 30-day readmission rates	Readmission rate by subgroup, time-to-event analysis, time to receive TCP components, and cost avoidance
Tedesco et al. [57]	USA	Prospective Quasi-experimental	Multi-site	Adults aged 65 years and over with Medicare insurance following hospital discharge	The number of patients readmitted to the same hospital within 30 days	
Westberg et al. [58]	USA	Prospective Quasi-experimental	Single	Adults aged 65 years or older and discharged from the hospital after being admitted for heart failure, ischeamic heart disease, dysrhythmias, genitourinary conditions, or digestive disorders	Hospital Readmissions	Emergency Department Visits Combined Medical Encounters, Mortality, and Adverse Events
Wiegmann et al. [48]	USA	Retrospective Observational	Single	Adults discharged within the last 14 days focussing on patients who; lacked insight into their chronic diseases, had significant changes in medications during their hospitalisation, or those hospitalised for diabetic ketoacidosis or hypertensive crisis	The rate of 90-day readmission for family medicine patients seen by a CP after hospital discharge compared to patients receiving standard of care	30- and 60-day readmission rates, reason for rehospitalization, time to readmission, time to initial post-discharge appointment, number of completed visits within the 90-day follow-up period, adherence to follow-up appointments, and attainment of chronic disease state goals

**Table 2:** Summary of concepts (nature of interventions) patterning chart

Summary of concepts (nature of interventions) patterning chart									
Author (First author), (Date)	Medication reconciliation	Medication review	Individualised care plan	Patient education and counselling	Liaising with other healthcare professionals	Review of laboratory monitoring	Medicines optimisation	Recommendations for laboratory work or referral for care co-ordination made	Long-term condition follow-up
Berquist et al. [39]	×	×			Hospital and care providers	×	×		
Brauner et al. [40]	×	×		×				×	
Cossette et al. [41]		×			Pharmacy and Clinicians				
Dellogono et al. [42]	×	×		×	Pharmacy				
Fera et al. [50]	×	×	×	×	Care providers				
Freeman et al. [43]	×	×			Pharmacy and Clinicians				
Haag et al. [49]		×			Care providers				
Hawes et al. [51]	×	×		×			×		
Herges et al. [53]	×	×							
Herges et al. [52]	×	×			Clinicians				
Herges et al. [44]	×	×			Clinicians				
Kilcup et al. [54]	×	×							
Ploenzke et al. [55]	×	×							
Shah C and Hough J and Jani [45]	×								
Slazak et al. [46]	×	×							
Sorensen et al. [47]	×				Care providers				
Stranges et al. [56]	×	×	×	×	Care providers		×		
Tedesco et al. [57]		×							
Westberg et al. [58]		×							
Wiegmann et al. [48]	×	×		×		×	×	×	×

Almost all studies showed that medication review (n = 18) [39–44, 46, 48–58] and medication reconciliation (n = 16) [39, 40, 42–56] formed the main interventions made by a primary care pharmacist post-hospital discharge. Emerging roles of primary care pharmacists such as liaising with other health care professional [39, 41–44, 47, 49, 50, 52, 56], medicines optimisation [39, 48, 51, 56] and review of laboratory monitoring [39, 48], referrals [40, 48] and follow-up [48] were also evident.

### Healthcare utilisation and readmission rates

The most frequently reported outcome was readmission rates, nine studies [40, 43, 47, 48, 53, 54, 56–58] reported readmission rates within a pre-specified time frame as their primary outcome; four studies [41, 43, 46, 53] reported readmission rates as a secondary outcome and two studies [52, 53] reported the risk of readmissions. Other studies reported on either acute care visits [50], emergency department visits or a combination to assess the impact of their intervention [41, 43, 47, 49, 51, 58]. Two studies [48, 56] reported on the time to readmissions following the intervention.

Not all studies reported the statistical significance of the outcomes, however, for those that did (n = 10) [41, 43, 44, 47, 48, 50, 54, 56–58], the results were inconsistent. Several studies showed a statistically significant reduction in ED presentation incidence [43] or combined readmission and ED presentation incidence [43, 47, 52, 54, 58]. In particular, the reduction in readmission rates was significant when completing the intervention, including a significantly longer time to readmission (18 ± 9 days compared with 12 ± 9 days with usual care; *P* = 0.015) [56]. Several other studies showed a non-statistically significant reduction in re-admission risk [52] or readmission rates post-hospital discharge [40, 43, 46, 48, 51, 56–58]. Furthermore, there was no statistically significant difference in outcomes when the pharmacist intervention is delivered face-to-face or over the telephone [57].

The key outcomes with statistically significant results [41, 43, 44, 47, 48, 50, 54, 56–58], were mapped to the intervention to review whether there was a common theme amongst them (Table 3). Overall, only ten of the 20 studies reported statistical significance in outcomes and all of those included medication review as part of the intervention. The medication review with a pharmacist varied between face-to-face or telephone medication review and all were conducted within 2 weeks of discharge from hospital varying between 2 and 14 days. The main outcomes where the significant impact was seen were categorised as a reduction in healthcare utilisation, reduction in hospital readmission rates, a longer time to readmission, improved clinical outcomes and beneficial economic impacts.

**Table 3:** Mapping of statistically significant outcomes against the PCP interventions

Outcome	Study	Intervention	Findings
*Reduction of healthcare utilisation*			
Reduction in ED presentation incidence during the 12 months following hospital discharge	Freeman et al. [43]	Comprehensive face-to-face medicine management consultation with an integrated practice pharmacist within seven days of discharge, followed by a consultation with their general practitioner and further pharmacist consultations as needed. (Medication review, medicines reconciliation, collaboration with other clinicians)	At 12 months, ED presentation incidence was 54% lower for intervention than control patients and the composite measure of re-admission and ED presentation incidence was 31% lower. ED presentation incidence and combined re-admission and ED presentation incidence were significantly lower for intervention patients
Reduction of acute care visits within 30 days	Fera et al. [50]	Care transitions Pharmacist (CTP) role, involving medication review, medication reconciliation, identification of MRPs, patient education, liaising with other care providers	Patients who received post discharge follow-up from the Care Transitions Pharmacist (CTP) were significantly less likely to have an acute care visit within 30 days of discharge compared with patients not contacted by the CTP
Combined medical encounters (hospital readmission and/or ED visit)	Westberg et al. [58]	Face-to-face medication review within 2 weeks of discharge	The number of combined medical encounters (hospital readmission and/or ED visit) during the 6 months following hospital discharge decreased significantly with time (discharge period), *P* < 0.05, but there was no significant intervention/ control difference in the number of encounters
*Reduction of readmission rates*			
Decreased readmission rates at 7 and 14 days	Kilcup et al. [54]	Telephone medication review 3–7 days post discharge	Decreased readmission rates at 7, 14, and 30 days post-discharge, with statistical significance at 7 and 14 days. This translates to one re-admission prevented for every 25 patients who receive clinical pharmacist medication therapy assessment and reconciliation
Reduction in readmission rates	Tedesco et al. [57]	Transition of care follow-up and counselling performed by a pharmacist, within a physician’s practice (medication review)	“Nearly a statistically significant decrease in readmission rates for those patients who interacted with the pharmacist face to face versus only telephonically (P = 0.05)”
Reduction in all-cause 30-day readmission rates	Wiegmann et al. [48]	Follow-up clinic appointment within 14 days post-discharge (medication review, medication reconciliation and chronic disease management)	All-cause 30-day readmission occurred significantly less often in patients followed by the CP
Reduction of readmission rates	Stranges et al. [56]	Primary care based transitional care program (pharmacist phone call 2–4 days after discharge -medication reconciliation, medication review) then seen in clinic within 1-week post-discharge by a social worker and medical provider, team approach)	When those completing the intervention (n = 217) were examined, readmission rates were significantly reduced (11.7% vs 17.3%, respectively; *P* < .001)
*Time to readmission*			
Time to Readmission	Wiegmann et al. [48]	Follow-up clinic appointment within 14 days post-discharge (medication review, medication reconciliation and chronic disease management)	Of the 100 patients included in the final analysis, time to readmission was longer in the intervention group versus standard of care group (52 vs 17 days, P = .026)
Time to Readmission	Stranges et al. [56]	Primary care based transitional care program (pharmacist phone call 2–4 days after discharge -medication reconciliation, medication review) then seen in clinic within 1-week post-discharge by a social worker and medical provider, team approach)	Time to readmission was significantly longer among those receiving the intervention (18 ± 9 days compared with 12 ± 9 days with usual care; *P* = .015)
*Clinical outcomes*			
Reduction in HgbA1c	Wiegmann et al. [48]	Follow-up clinic appointment within 14 days post-discharge (medication review, medication reconciliation and chronic disease management)	The average reduction in HgbA1c for patients with diabetes from discharge through the end of the 90-day follow-up period was greater in the CP intervention group versus the standard of care group
*Cost*			
Improvement in total cost of care in medically complex patients	Herges et al. [44]	Medication Review—PCC (Pharmacist-clinician collaborative- (30-min visit with a pharmacist, followed immediately by a 30-min visit with a primary care clinician)	Medically complex patients had a significantly lower total cost of care in approximately half of the adjusted cost quantiles at 30, 60, and 180 days after hospital discharge when they had a Pharmacist/Physician Collaborative (PCC) visit compared to usual care
Cost benefit of the intervention	Freeman et al. [43]	Comprehensive face-to-face medicine management consultation with an integrated practice pharmacist within seven days of discharge, followed by a consultation with their general practitioner and further pharmacist consultations as needed. (Medication review, medicines reconciliation, collaboration with other clinicians)	Estimates net cost benefit of the intervention was $5072 per patient, with a benefit–cost ratio of 31:1
Financial savings	Kilcup et al. [54]	Telephone medication review 3–7 days post discharge	Financial savings for Group Health per 100 patients who received medication reconciliation was an estimated $35,000, translating to more than $1,500,000 in savings annually
Cost avoidance when intervention was completed	Stranges et al. [56]	Primary care based transitional care program (pharmacist phone call 2–4 days after discharge -medication reconciliation, medication review) then seen in clinic within 1-week post-discharge by a social worker and medical provider, team approach)	Potential cost avoidance was observed only when the intervention was completed. Based on results of the as-treated analysis, Hospitalisation cost avoidance was estimated to be $737,673 among the 345 completed interventions, or $2138 per intervention

### Economic implications

From the 20 studies, eight studies (40%) reported on the financial impact of the intervention [39, 43, 44, 50, 51, 54, 56, 57]. However, only four studies (20%) evaluated the actual cost-savings [43, 44, 54, 56]. Evidence showed that there was a significantly lower total cost of care after pharmacist-clinician collaborative visits whilst also improving clinical outcomes [44]. One study showed an estimated incremental net benefit of $5054 per patient and after sensitivity analysis translated a benefit–cost ratio of 28:1 [43]. Kilcup et al. [54] estimated cost savings per 100 patients to be $35,000, equivalent to $1,500,000 in savings annually.

### Collaborations with other healthcare professionals

**Fig. 2:**
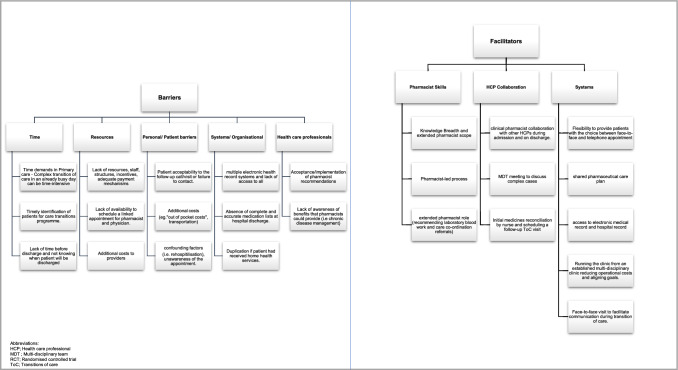
Barriers and facilitators to PCP interventions

Seventeen studies reported collaborations of primary care pharmacists with other healthcare professionals [39–44, 47–53, 55–58]. Contact was made with the hospital inpatient team or hospital pharmacists if the information was missing or required clarification. Often, where further support or supervision was required or the task was outside the pharmacists’ scope of practice, patient’s primary care physician was contacted through verbal, written or electronic approaches [42, 52, 53, 55]. In some studies, a physician appointment immediately followed an appointment with the PCP [43, 44, 48, 51–53]. A team approach was often advocated in medicines-management during transitions of care [42, 48, 50, 56, 58]. In addition to collaboration between pharmacist and physicians, communication with community-based health coaches [47], care co-ordinators [40, 58] and other healthcare providers [39, 42, 43, 51, 55, 58, 59] were also reported (Fig. 2).

### Barriers and facilitators to PCP interventions

The main barriers identified in the studies were time demands in an already busy healthcare environment and lack of resources to provide the timely intervention. Most studies reported time to intervention of between three to seven days [39, 51, 52, 54, 56]. The time to deliver the intervention (clinic medication review with the pharmacist and documentation) substantially varied between studies and took between 45 and 90 min [39–41, 55]. Other key barriers reported were: acceptability of the intervention by the patients [49, 50, 56], healthcare professionals’ acceptability of recommendations [50, 53] and lack of awareness of the role or benefits of pharmacists [50, 53]. The potential financial impact on patients [54, 57] and lack of adequate payment mechanisms/incentives within organisations [50, 56], were also highlighted as possible barriers to interventions. In addition, systems and organisational barriers were reported which can be confounded by lack of accessibility to multiple electronic systems and accurate medication lists at discharge [40, 51].

Facilitators for the intervention were the value that the pharmacist skills and roles can bring and the collaboration with other healthcare professionals [40, 41]. This was recognised for complex cases during a multi-disciplinary team meeting and having a shared pharmaceutical care plan [41]. This shared care plan would enable continuity and easier access across sectors in addition to running the clinic from an established multi-disciplinary clinic to align care goals and also reduce costs [56]. One study by Slazak et al. [46] reported that having a registered nurse contact patients and complete the initial medicines reconciliation and then scheduling a follow-up visit with the pharmacist was a facilitator in the process. Furthermore, the flexibility to provide patients with a choice between face-to-face and telephone appointment was seen as a facilitator, although face-to-face visits assisted communications during transitions of care [40].

## Discussion

This is the first review, to the authors’ knowledge, that maps the extent and nature of the role of PCPs in a general practice setting, specifically post-hospital discharge. The contributions of pharmacists in the transition from secondary to primary care are heterogenous and outcome success rates have been variable. Evidence has shown that both medication review and medication reconciliation feature frequently as part of the PCP intervention which corresponds with evidence about pharmacists’ roles more widely [16–18, 24, 63]. The PCP role is evolving to include other interventions such as: monitoring of laboratory results; management of long-term conditions and generating referrals to other health care professionals. There is growing evidence about the value of multi-disciplinary team approaches and collaborations with other practitioners to enable better patient care [25, 27] and the continued need for improved communication between secondary and primary care during transition processes [66]. We have identified the emerging of the role of the PCP to date, however there are many areas that require further research as outlined in our PAGER framework (Table 4).

**Table 4:** PAGER framework analysis

Patterns	Advances	Gaps	Evidence for practice	Research recommendations
The nature of pharmacist-led intervention in primary care primarily includes medication review and medication reconciliation	There is an emerging role of collaborating and liaising with other pharmacy teams, physicians, and care providers to improve care. In addition, primary care pharmacists are actively involved in optimising medication and individualising patient care	There is a variation in scope of practice and skill set of primary care pharmacists. For example, some may be prescribers and able to manage specific conditions and some may need to refer to other clinicians e.g., for laboratory monitoring. The impact on this is not clear in current studies	There is an advancement in the scope of practice of pharmacists around the world and we do not know the impact of this yet on improving medication management post-hospital discharge	More contemporary research is required to investigate the scope and impact of primary care pharmacists given the evolving roles and changes happening in health and social care systems, e.g. the move towards integrated care systems in the United Kingdom
Location: The large body of published studies is mainly from the USA	Further studies are being published in other countries such as Australia and the UK as the role of primary care pharmacists evolves and expand	There is a paucity of UK studies in this sector. There are also differences in the healthcare systems in different countries therefore evaluations across countries may not be generalisable	Interventions and models of care exist and are delivered within a complex health and social care system. The dynamics between the context and the intervention or models of care are important for understanding what is (or is not) working, how, and why	Further studies on the impact of primary care pharmacists in other countries would be valuable to improve understanding of context, and how the intervention works in that context towards achieving intended and unintended outcomes. This will provide richer, more nuanced information about the roles and impact of PCPs at the transitions of care
Study methods: Quantitative methods	Some studies have discussed patient satisfaction surveys in addition	Little research of a qualitative nature has been undertaken on the role of primary care pharmacists post-hospital discharge	Evidence has shown that primary care pharmacists have a role to play at transitions of care and this has been validated by multiple quantitative studies mainly in the USA	Patient and healthcare professionals’ perceptions of experiences with medication management post-hospital discharge would be valuable to understand the potential roles of PCPs Furthermore, more studies that have proxy measures such as adherence may have the potential to validate any causal pathways. Other studies in patients who have poor health literacy or are more disadvantaged would be valuable
Most studies have focused on outcomes such as hospital re-admission rates and reduction of healthcare utilization	Previous research has shown that pharmacists have a key role in identifying medication-related problems and medicines optimisation, however, we now know that pharmacists can contribute to long-term condition management and use their expanded clinical skills within a multi-disciplinary team	There is sparse evidence about the impact of PCP involvement on clinical outcomes post-hospital discharge	Hospital readmission rates and healthcare utilization are relatively downstream impacts of a post-discharge intervention and are often challenging to attribute to a singular intervention. Impact on more proximal clinical outcomes would be more helpful to explore causal relationships between an intervention and an outcome	More high-quality experimental/quasi-experimental research to investigate the impact of PCP involvement in transitions of care on clinical outcomes and humanistic outcomes would be a significant contribution to inform policy and practice

Whilst it is known generally that pharmacists can have positive impacts on patients’ understanding of medications [60, 61], patient satisfaction [26] and clinical outcomes [20], few studies have looked at this in relation to PCPs role post-hospital discharge [48] and this would complement current research. Furthermore, the role of PCP involvement in motivational interviewing and adherence strategies [50] to reduce medication-related problems post-hospital discharge would be useful, especially in those cohorts who may experience poor health literacy or barriers to medication adherence. Few studies have evaluated economic impacts [43, 44, 54, 56] of PCPs post-hospital discharge, however these have reported beneficial impacts at reducing costs indirectly as some health systems have applied payment penalties for high 30-day readmission rates [50, 51, 57, 62]. These indirect cost implications and benefits of PCP involvement post-hospital discharge and in longer-term management should be further explored relevant to the respective healthcare system or certain cohorts of patients that would benefit (i.e. particular health conditions, high-risk medicines or those with specific needs like compliance aids). The lack of controlled studies and consistency in outcomes researched highlights the need for further high-quality research in this field to develop robust transition of care pathways relevant to local healthcare systems. Additionally, studies on a larger scale in different geographical locations to support wider generalisability and transferability of findings would be valuable. The identified barriers and facilitators are those commonly reported when considering the implementation of clinical services by pharmacists in primary care, including barriers to integration and lack of interoperability of digital systems [63]. Further systematic investigation of factors influencing practice would be helpful, particularly using lenses of behavioural and implementation science. This would enable designing or optimising future models of care or interventions that address the barriers and capitalise on the facilitators.

### Limitations

The scoping review intended to map the evidence in this field, therefore does not allow for meta-analysis or critical appraisal of the effectiveness of the interventions studied. During the scoping review, conference abstracts and studies relating to home-visits were excluded as per the protocol which may have limited our findings. As this review was primarily conducted to inform further research relevant to the UK population, a grey literature search of only UK professional organisations and websites was a limitation as we did not search for grey literature in other countries. Furthermore, the grey literature found articles that were not eligible due to the strict nature of the inclusion and exclusion criteria in the protocol relating to publication type and primary studies of an experimental or observational nature. This may have limited any audit, evaluation studies or case reports that have been carried out in clinical practice.

## Conclusion

The scoping review allowed valuable mapping of the extent of research carried out on the role of primary care pharmacists during post-hospital discharge. It is apparent that there is an evolving scope of practice which could prove valuable to a primary care based multi-disciplinary team with positive effects on patient and healthcare utilisation outcomes. Our findings highlight the gaps in evidence to date to help inform future priorities and directions of research in this area, identifying that more rigorous research is needed to establish effectiveness and generalisability of primary care pharmacist interventions.

## Supplementary Information

Below is the link to the electronic supplementary material.

Supplementary file1 (DOCX 22 KB)

Supplementary file2 (DOCX 19 KB)
