# Supplementary material for: Facilitating the transfer of care from secondary to primary care: a scoping review to understand the role of pharmacists in general practice
Source: Int J Clin Pharm. 2023 Mar 15;45(3):587–603. doi: 10.1007/s11096-023-01547-3 (PMC10016159; doi:10.1007/s11096-023-01547-3)
Supplement: Supplementary file 1 — Supplementary file1 (DOCX 22 KB) [file 11096_2023_1547_MOESM1_ESM.docx]

| **Database** | **Search Terms** | **Date Search Run** | **Number of results** |
| --- | --- | --- | --- |
| **Ovid MEDLINE(R) and In-Process, In-Data-Review & Other Non-Indexed Citations <1946 to March 17, 2022>** | 1 (Pharmacist* adj5 ("primary care" or clinical or "practice based" or "general practi*" or "GP practice*" or prescrib* or "non-dispensing")).mp. [mp=title, abstract, original title, name of substance word, subject heading word, floating sub-heading word, keyword heading word, organism supplementary concept word, protocol supplementary concept word, rare disease supplementary concept word, unique identifier, synonyms] 7420  2 Patient Discharge/ or "post-hospital discharge".mp. 35832  3 Patient Discharge/ or "post-hospitalisation".mp. or Hospitalization/ 157179  4 Hospitalization/ or Patient Discharge/ or "post hospitalisation".mp. 157179  5 Hospitalization/ or Patient Discharge/ or "post-hospitalization".mp. 157428  6 Hospitalization/ or Medication Reconciliation/ or "medic* reconciliation".mp. 127984  7 Patient Transfer/ or "transfer of care".mp. 9853  8 Patient Discharge/ or "Continuity of Patient Care"/ or Patient Transfer/ or Transitional Care/ or "care transition*".mp. 63266  9 "TCAM".mp. 177  10 2 or 3 or 4 or 5 or 6 or 7 or 8 or 9 186087  11 1 and 10 693  12 limit 11 to english language 672 | 18.3.22 | 672 |
| **Embase <1974 to 2022 March 16>** | 1 (Pharmacist* adj5 ("primary care" or clinical or "practice based" or "general practi*" or "GP practice*" or prescrib* or "non-dispensing")).mp. [mp=title, abstract, heading word, drug trade name, original title, device manufacturer, drug manufacturer, device trade name, keyword heading word, floating subheading word, candidate term word] 16858  2 *hospital discharge/ or "post hospital discharge".mp. 15539  3 "post hospitalisation".mp. or *hospital discharge/ 14985  4 *hospital discharge/ or "post hospitalization".mp. 15928  5 *medication reconciliation/ or "medic* reconciliation".mp. 7471  6 "transfer of care".mp. 1214  7 "care transition*".mp. 3520  8 "TCAM".mp. 258  9 2 or 3 or 4 or 5 or 6 or 7 or 8 28159  10 1 and 9 1390  11 limit 10 to english language 1337 | 18.3.22 | 1337 |
| PubMed | ("pharmacist*"[Title/Abstract] AND ("primary care"[Title/Abstract] OR "clinical"[Title/Abstract] OR "practice based"[Title/Abstract] OR "general practi*"[Title/Abstract] OR "gp practice*"[Title/Abstract] OR "prescrib*"[Title/Abstract] OR "non-dispensing"[Title/Abstract]) AND "english"[Language] AND (("Post hospital discharge"[Title/Abstract] OR "post hospitalisation"[Title/Abstract] OR "post hospitalization"[Title/Abstract] OR "medicines reconciliation"[Title/Abstract] OR "transfer of care"[Title/Abstract] OR "care transition*"[Title/Abstract] OR "TCAM"[Title/Abstract]) AND "english"[Language])) AND (english[Filter])  *As PubMed was failing to recognise N5 and “medic* reconciliation”* | 18.3.22 | 170 |
| Cochrane central register of controlled trials (CENTRAL) | #1 Pharmacist* adj5 ("primary care" or clinical or "practice based" or "general practi*" or "GP practice*" or prescrib* or "non-dispensing") 135  #2 "post hospital discharge" 232  #3 "post hospitalisation" 218  #4 "post hospitalization" 218  #5 "medic* reconciliation" 0  #6 "transfer of care" 52  #7 "TCAM" 8  #8 #2 OR #3 OR #4 OR #5 OR #6 OR #7 501  #9 #1 AND #8 **6**  *Cochrane reviews=6* | 18.3.22 | 0 |
| Web of Science | **#1 AK=(Pharmacist* AND("primary care" or clinical or "practice based" or "general practi*" or "GP practice*" or prescrib* or "non-dispensing")) = 1864**  **#2 AK=("Post hospital discharge" OR "post hospitalisation" OR "post hospitalization" OR "medic* reconciliation" OR "transfer of care" OR "care transition*" OR "TCAM")**  =1660  #1 AND #2 = 40  Limit - English Language = 38 | 18.3.22 | 38 |
| NICE Evidence. | (Pharmacist* AND("primary care" OR clinical OR "practice based" OR "general practi*" OR "GP practice*" OR prescrib* OR "non dispensing")) AND ("Post hospital discharge" OR "post hospitalisation" OR "post hospitalization" OR "medic* reconciliation" OR "transfer of care" OR "care transition*" OR "TCAM") *Total 224*  *Guidance and Policy 77*  *Secondary evidence 66*  *Primary Research 48* | 18.3.22 | 48 |

| **Websites of Relevant Professional Organisations searched** |  |  |  |
| --- | --- | --- | --- |
| Royal Pharmaceutical Society | Multiple combinations attempted- 0 results  Also searched Pharmaceutical Journal = 0 (Pharmacist* AND("primary care" OR clinical OR "practice based" OR "general practi*" OR "GP practice*" OR prescrib* OR "non dispensing")) AND ("Post hospital discharge" OR "post hospitalisation" OR "post hospitalization" OR "medic* reconciliation" OR "transfer of care" OR "care transition*" OR "TCAM")(Pharmacist* N5 "primary care" OR clinical OR "practice based" OR "general practi*" OR "GP practice*" OR prescrib* OR "non dispensing")) AND ("Post hospital discharge" OR "post hospitalisation" OR "post hospitalization" OR "medic* reconciliation" OR "transfer of care" OR "care transition*" OR "TCAM")  (Pharmacist* AND ("Post hospital discharge" OR "post hospitalisation" OR "post hospitalization" OR "medic* reconciliation" OR "transfer of care" OR "care transition*" OR "TCAM") Pharmacist AND "post-hospital discharge" | 20.3.22 | 0 |
| General Pharmaceutical Council | Primary care pharmacist AND post hospital discharge  OR (pharmacist AND transfer of care)  (Documents and Regulate Articles) = 174  Filter by document category - consultations and policy papers, corporate publications, guidance, reports =124 | 20.3.22 | 124  (2 potentially eligible) |
| Royal College of General Practitioners | Primary care pharmacist AND post hospital discharge =0  Pharmacist* AND post hospital discharge = 0  Pharmacist AND post hospital discharge = 0  pharmacist AND transfer of care= 0 | 20.3.22 | 0 |
| Department of Health | (Pharmacist* N5 "primary care" OR clinical OR "practice based" OR "general practi*" OR "GP practice*" OR prescrib* OR "non dispensing")) AND ("Post hospital discharge" OR "post hospitalisation" OR "post hospitalization" OR "medic* reconciliation" OR "transfer of care" OR "care transition*" OR "TCAM")) | 20.3.22 | 3 |
| The UK faculty of public health website and the Journal of Public Health | (Pharmacist* AND("primary care" OR clinical OR "practice based" OR "general practi*" OR "GP practice*" OR prescrib* OR "non dispensing")) AND ("Post hospital discharge" OR "post hospitalisation" OR "post hospitalization" OR "medic* reconciliation" OR "transfer of care" OR "care transition*" OR "TCAM") Pharmacist AND post hospital discharge= 0  Pharmacist AND transfer of care = 0 | 20.3.22 | 0 |
| National Institute for Health and Care Excellence (NICE) | (Pharmacist* AND("primary care" OR clinical OR "practice based" OR "general practi*" OR "GP practice*" OR prescrib* OR "non dispensing")) AND ("Post hospital discharge" OR "post hospitalisation" OR "post hospitalization" OR "medic* reconciliation" OR "transfer of care" OR "care transition*" OR "TCAM") =14 Pharmacist AND post hospital discharge = 36  Pharmacist AND transfer of care =14 | 20.3.22 | 36  (9 potentially eligible) |
