# Supplementary material for: Facilitating the transfer of care from secondary to primary care: a scoping review to understand the role of pharmacists in general practice
Source: Int J Clin Pharm. 2023 Mar 15;45(3):587–603. doi: 10.1007/s11096-023-01547-3 (PMC10016159; doi:10.1007/s11096-023-01547-3)
Supplement: Supplementary file 2 — Supplementary file2 (DOCX 19 KB) [file 11096_2023_1547_MOESM2_ESM.docx]

**Supplementary File 2**

**Search terms**

For comprehensiveness and scoping, the search terms were kept broad in nature and adapted for each source using truncation, Boolean operators, and medical subject headings as appropriate.

The two concepts for the research question were combined with ‘AND’:

**Concept 1:** Pharmacist* adj5 (“primary care” OR clinical OR “practice based” OR “general practi*” OR “GP practice*” OR prescrib* OR “non-dispensing”)

**Concept 2:** “Post hospital discharge” OR “hospital discharge” OR “post hospitalisation” OR “post hospitalization” OR “medic* reconciliation” OR “transfer of care” OR “care transition*” OR “TCAM”
